# New Horizons: Novel Adrenal Regenerative Therapies

**DOI:** 10.1210/clinem/dgaa438

**Published:** 2020-07-06

**Authors:** Stefan R Bornstein, Maria Malyukov, Carolin Heller, Christian G Ziegler, Gerard Ruiz-Babot, Andreas Schedl, Barbara Ludwig, Charlotte Steenblock

**Affiliations:** 1 Department of Internal Medicine III, University Hospital Carl Gustav Carus, Technische Universität Dresden, Dresden, Germany; 2 Diabetes and Nutritional Sciences Division, King’s College London, London, UK; 3 Paul Langerhans Institute Dresden (PLID) of the Helmholtz Center Munich, University Hospital Carl Gustav Carus, Technische Universität Dresden, Dresden, Germany; 4 DFG-Center for Regenerative Therapies Dresden, Technische Universität Dresden, Dresden, Germany; 5 Division of Endocrinology, Boston Children’s Hospital, Boston, Massachusetts, USA; 8 Department of Pediatrics, Harvard Medical School, Boston, Massachusetts, USA; 6 Université Côte d’Azur, INSERM, CNRS, iBV, Nice, France; 7 Department of Endocrinology and Diabetology, University Hospital Zurich, Zurich, Switzerland

**Keywords:** adrenal, stem cells, adrenal insufficiency, cell replacement, gene therapy, regenerative therapies

## Abstract

Adrenal insufficiency requires lifelong corticoid replacement therapies. However, current therapies are not able to replace the physiological circadian pattern of the adrenal cortex and are associated with many metabolic, vascular, neuroendocrine, and mental perturbations. Therefore, regenerative and more curative strategies would be desirable. In the current perspective, we describe emerging new regenerative therapies for the treatment of adrenal insufficiency. In particular, we discuss gene therapy and cell replacement strategies. Furthermore, we discuss how adrenal cells might be used as a source for regenerative therapies of nonadrenal neurodegenerative diseases such as Parkinson disease.

The adrenal gland displays an important role in integrating neuronal, immune, vascular, metabolic, and endocrine signals. The adrenal represents the central organ in the stress response system and coordinates reactions to various acute and chronic stress stimuli. Thereby, the adrenal gland plays a major role in numerous stress-related disorders. Diseases of the adrenal gland itself lead to dysfunction of different parts of this complex endocrine unit and may cause hyper- or hypofunctional conditions that are either congenital or acquired. To recognize how regenerative therapies might help treating adrenal hypofunction, it is important to understand normal adrenal development and regeneration.

## Adrenal Development and Regeneration

The adrenal gland is composed of 2 main tissue types that establish a bidirectional connection: the catecholamine-producing chromaffin cells in the medulla and the primarily steroid-producing cells in the cortex. Close interactions between these 2 components are necessary for differentiation and morphogenesis of the gland. The adrenal cortex consists of 3 different zones: the outer zona glomerulosa, the zona fasciculata, and the zona reticularis, which surrounds the medulla. Each of these zones produces distinct steroid hormones (mineralocorticosteroids, glucocorticoids, and androgens, respectively), which are involved in a variety of physiological functions (1). In mice, the zona reticularis is absent, but during certain postnatal developmental stages an X-zone, derived from the fetal adrenal, is present.

In the adult adrenal gland, different populations of progenitor cells, residing in the adrenal capsule, cortex, and medulla, have been described (1, 2). Homeostasis and regeneration in the adrenal cortex are maintained through capsular and cortical progenitors (1). In response to paracrine and endocrine signaling these cells proliferate, migrate centripetally, and differentiate into cortical steroid-producing cells. In mice, cortical cell renewal appears to be sex-dependent, and females show a 3-fold higher tissue turnover than males. This sexual dimorphism is under hormonal control, and it has been shown that androgens suppress the recruitment of capsular cells to the steroidogenic lineage and inhibit proliferation of steroidogenic cells (1). In vitro, testosterone has been shown to affect proliferation of human mesenchymal stem and endothelial progenitor cells, but if androgens also have an effect on adrenocortical cell turnover in humans still needs to be elucidated. In the adrenal medulla, progenitor cells give rise to neurons, glia and chromaffin cells.

## Adrenal Insufficiency

Adrenal insufficiency is a condition in which the adrenal gland does not produce adequate amounts of glucocorticoids and/or mineralocorticoids. These steroids play a central role in the body’s homeostasis of energy, salt, and fluids; thus, adrenal insufficiency is a potentially life-threatening condition. Primary adrenal insufficiency is due to impairment of the adrenal gland with ~80% of the cases being from autoimmune adrenalitis (Addison disease). Other cases of primary adrenal insufficiency might be idiopathic, caused by adrenal metastases, or due to congenital adrenal hyperplasia (CAH). CAH is a group of inherited autosomal recessive disorders encompassing deficiencies in the adrenal steroidogenesis pathway leading to impaired cortisol biosynthesis with 21-hydroxylase deficiency due to genetic changes in the *CYP21A* gene as the most common cause.

In addition, adrenal insufficiency can be induced by infectious diseases and, as seen lately in an increasing number of patients, by novel medications targeting hypertension and cancer. For example, steroid hormone synthesis inhibitors or immune checkpoint inhibitors lead to an impairment of the hypothalamic-pituitary-adrenal (HPA) axis. Last, surgical bilateral adrenalectomy as required in certain adrenal tumors causes complete adrenal insufficiency, necessitating an effective hormone substitution therapy.

Despite decades of evaluating various treatment algorithms, the management of adrenal insufficiency remains a major therapeutic challenge (3). Pharmacological corticosteroid substitution is a lifesaving procedure; however, it cannot restore physiological feedback regulation of an intact HPA axis, the essential circadian and pulsatile hormone secretion is not to be mimicked, and physiologically necessary adaptations to variable demands are not automatically happening. Moreover, there is a high incidence of side effects from inappropriate glucocorticoid substitution. Drastic therapeutic measures such as bilateral adrenalectomy are effective to treat female infertility of CAH patients but, because of the risk of adrenal crisis, this option is only considered in patients in whom other treatments have failed (4). Prenatal therapy with steroids such as dexamethasone may also bear particular risks. Current emerging therapeutic alternatives include new and more physiological means of glucocorticoid delivery, inhibitors, or antagonists at the level of CRH or adrenocorticotropic hormone (ACTH) secretion and/or action, as well as gonadotropin-releasing hormone analogs, anti-androgens, aromatase inhibitors, and estrogen receptor blockers. Many of these new treatment options show promising results in preclinical studies, but still require additional clinical validation. For example, different approaches where the glucocorticoid concentration has been optimized through delayed release do show an improved cortisol pattern, but cannot fully recapitulate the normal physiological circadian rhythm (4).

## Restoration of Residual Adrenal Function

Autoimmune Addison disease is generally regarded as an irreversible progressive disease in which the adrenal gland is destroyed. However, numerous case reports have shown that in some patients, spontaneous remission has occurred. This suggests a high degree of heterogeneity in adrenal function in patients with Addison disease. Furthermore, recent studies have shown that shortly after the onset of Addison disease, residual adrenal function might be restored using B-lymphocyte-depleting immunotherapy and ACTH treatment (5). Because the autoimmune response is directed against steroidogenic enzymes expressed in adrenocortical cells and not in the adrenocortical stem cells, ACTH might stimulate the differentiation of the persisting progenitor or stem cells and, in combination with the B-cell depletion, the newly differentiated steroidogenic cells will not be destroyed by the immune cells. Currently, success using this strategy is limited with only small serum cortisol and urine steroid metabolite increases. Nevertheless, this therapy has the potential to at least improve adrenal function.

## Gene Therapy

Gene therapy might be a possibility for restoring steroidogenesis in CAH, which is a monogenic disease. Approaches in this direction ~20 years ago based on adenovirus-mediated gene transfer of human *CYP21A* into 21-hydroxy-deficient mice indeed showed improvements in animal models of CAH. Because of various functional limitations and regulatory restrictions, these approaches were not processed beyond the early stages. Nevertheless, very recently, several research groups have taken up this idea again, and it was shown that transfer of the human *CYP21A* gene to mouse models of CAH ameliorates the systemic steroid metabolism. These promising results suggest that gene therapy could in fact be a feasible option for treatment of CAH. However, 1 limitation is that the effect seems to be only temporary because of cortical cell turnover (6). This means that achieving a long-term correction requires that the transgene is inserted into the genome of adrenocortical stem cells.

## Cell Replacement Strategies

Adrenal cell transplantation would be a desirable therapeutic alternative for patients with adrenal insufficiency. Such strategy would go beyond the evident goal of replacing insufficient hormones and rather allow for restoration of the HPA axis function and a possible curative strategy.

Cellular therapies aim to repair the mechanisms underlying disease initiation and progression, and such therapies are designed for various disorders including cardiovascular, neurological, autoimmune, and others. Multiple cell types can be used in these therapies, including primary, stem, or progenitor cells. For pancreatic islets, significant success has been achieved in replacement strategies for the treatment of type 1 diabetes, and human islet transplantation has become an established therapy for patients with type 1 diabetes. However, because of the general limitations of human allogeneic cell and organ replacement strategies, alternative cell sources such as xenogeneic cells are being generated to form the basis for cell replacement strategies of endocrine tissues. The availability of such alternative cell sources has generated a renewed attention for macroencapsulation strategies as a safeguard to control the potential risks of inflammatory reactions and graft rejection. Furthermore, endocrine tissues such as pancreatic islets and the adrenal are complex multicellular functional entities that are highly dependent upon their specific microenvironment. To overcome these limitations, optimization of adrenal transplantation and use of immuno-isolating biomaterials and/or devices has been initiated. In this regard, novel biomaterials have been developed to improve cell viability of engineered cells upon implantation. Biomaterials are mostly based on the principle of biomimicry and aim to resemble the extracellular matrix around the cells to establish a niche that supports cell viability and function. In their natural niche, cells are interacting with the extracellular matrix consisting of glycosaminoglycans and proteins. These support the cells with anchor points where the cells can attach, as for example integrin binding motifs on fibronectin or laminin. In the biomaterial field, the extracellular matrix is mimicked by natural or synthetic polymers. These can often take up large amounts of water establishing a 2-dimensional or 3-dimensional hydrogel environment for the desired cells. Some of these polymers also give the possibility to release growth factors or establish gradients for cell differentiation. An approach for the differentiation of steroid-producing cells could be to place progenitor cells into such hydrogels, which have an adapted stiffness similar to the adrenal. Furthermore, anchor points could be provided that are establishing steroid-producing cell specific cell-matrix interactions. A variety of systems has been reported that implement novel customized polymers that recapitulate key properties of the endocrine environment and therefore may promote survival and function, reduce inflammatory reactions, and, by barrier-creating methodologies, might allow for transplantation in the absence of immunosuppression. For example, our group has developed a macroencapsulation system, originally designed for pancreatic islet encapsulation that provides sufficient immune-isolation while maintaining regular cell function. This concept has been tested successfully in various preclinical models of diabetes, in a first clinical case of type 1 diabetes and has now entered the clinical trial application phase. Based on this experience, this concept has been adopted for the development of an adrenal cell replacement therapy, the ultimate alternative for patients suffering from adrenal insufficiency. Thereby, bovine adrenocortical cells embedded in alginate and encapsulated in an immunoisolating device were transplanted into adrenalectomized rats. This procedure was shown to be an effective method to avoid the need of chronic immunosuppression (7). Furthermore, this model provided a microenvironment that ensured 3-dimensional cell–cell interaction and where fibrosis was suppressed. The transplants were highly vascularized and viable for at least 4 weeks. For encapsulated adrenocortical cells, the cortisol production was highly increased when compared with transplantation of nonencapsulated cells (7). Approaches to optimize the viability have shown that the presence of stem cells increases the functionality of the transplant. Thereby, instead of using whole organs or fully differentiated adrenocortical cells, stem cell therapy is an additional option. Stem cell therapy has evolved from the early stages of using embryonic cells to advanced applications of attempting to repair and restore human organs. Pluripotent stem cells (PSCs) offer the possibility of an unlimited, renewable source of replacement cells and tissues to restore adrenal functionality. Cellular therapies based on PSCs and cell reprogramming techniques have been developed for different tissues; however, such approaches have been largely overlooked in the adrenal field. Different cell types have been used as a substrate to generate cells with steroidogenic potential (3). Adult stem cells from different sources, such as urine, adipose tissue, bone marrow, umbilical cord, or blood have been successfully reprogrammed to steroid-producing cells by overexpressing SF-1/Ad4BP. Embryonic stem cells or induced PSCs, which have the potential to generate all cell types in the body, have also been differentiated toward a steroidogenic phenotype upon gene manipulation and/or modulation of specific pathways. Such cells express steroidogenic enzymes and are able to produce a range of different steroid hormones in vitro. However, only limited in vivo studies have been performed using reprogrammed mouse or human cells in the adrenal field. Although cells are viable after implantation into mice, full functionality and responsiveness to adrenal stimuli have not been reported. These observations might be due to incomplete differentiation and limited steroidogenic potential of the cells before transplantation. Therefore, another possibility is to generate steroid-producing cells directly from adult adrenocortical stem cells/progenitors (2). Thereby, cell replacement therapies involving donor-derived stem cells or patient-derived stem cells, in which a specific mutation has been corrected, would represent an attractive alternative. Looking forward, the generation of bona fide steroidogenic cells from humans combined with novel biomaterials and encapsulation in immune-isolating devices might offer alternative therapies for patients with adrenal insufficiency.

## Non-Adrenal Diseases

Cellular replacement strategies using adrenal cells have also been considered for other diseases than adrenal insufficiency. Since the 1980s, different research groups have tested the possibility of using chromaffin cells for the treatment of pain because these cells produce several neuroactive substances, including catecholamines and opioid peptides, which can cause pain relief. Although these strategies are still in their infancy phase for pain neurorestoratology, cell-based therapies could open up new avenues for the relief of pain.

Chromaffin cells from the adrenal medulla have also been considered as a potential source of dopamine-producing cells to treat neurodegenerative conditions like Parkinson disease (PD) (8). The physiopathology of PD is associated with the loss of dopaminergic neurons, and because of the close relation of chromaffin cells to catecholaminergic neurons, a substantial number of studies has promoted the use of chromaffin cells for this purpose. However, transplantation of chromaffin cells in patients produced only partial motor improvements and these were only transient and highly variable among subjects. In addition, high morbidity and mortality were associated with this grafting procedure. Therefore, within the past few years, adrenomedullary stem cells have been tested for cell replacement therapies of PD. Following this strategy, a significantly higher population of cells was shown to acquire a dopaminergic phenotype when compared with fully differentiated chromaffin cells.

## Conclusion

Current treatments for adrenal insufficiency are limited to glucocorticoid, mineralocorticoid, and DHEA replacement strategies. Glucocorticoids are secreted following a circadian rhythm, which is impossible to adequately recapitulate using current replacement therapies with synthetic glucocorticoids; therefore, patients often suffer from poor quality of life and increased mortality. Promising results have recently been obtained using cell replacement therapies. In particular, encapsulation of adrenocortical (stem) cells opens new prospects for successful transplantation. Furthermore, stem cells from the adrenal medulla might have the potential to be used for the treatment of neurodegenerative diseases. However, more studies and optimization are needed before a long-term functioning transplantable graft is available for the treatment of adrenal insufficiency or non-adrenal diseases using adrenal cells and stem cells. Furthermore, such an ambitious endeavor obligatory requires a high level of interdisciplinarity including physicians, cell biologists, immunologists, and materials scientists.

## Abbreviations

ACTH: adrenocorticotropic hormone
CAH: congenital adrenal hyperplasia
HPA: hypothalamic-pituitary-adrenal
PD: Parkinson disease
PSC: pluripotent stem cell

## Additional Information

***Disclosure Summary:*** The authors have nothing to disclose.

***Data Availability:*** Data sharing is not applicable to this article as no datasets were generated or analyzed during the current study.
